# Contrasting *in vitro* and *in vivo* methanol oxidation activities of lanthanide-dependent alcohol dehydrogenases XoxF1 and ExaF from *Methylobacterium extorquens* AM1

**DOI:** 10.1038/s41598-019-41043-1

**Published:** 2019-03-12

**Authors:** Nathan M. Good, Riley S. Moore, Carly J. Suriano, N. Cecilia Martinez-Gomez

**Affiliations:** Martinez-Gomez laboratory, Department of Microbiology and Molecular Genetics, Michigan State University, East Lansing, MI USA

## Abstract

Lanthanide (Ln) elements are utilized as cofactors for catalysis by XoxF-type methanol dehydrogenases (MDHs). A primary assumption is that XoxF enzymes produce formate from methanol oxidation, which could impact organisms that require formaldehyde for assimilation. We report genetic and phenotypic evidence showing that XoxF1 (MexAM1_1740) from *Methylobacterium extorquens* AM1 produces formaldehyde, and not formate, during growth with methanol. Enzyme purified with lanthanum or neodymium oxidizes formaldehyde. However, formaldehyde oxidation via 2,6-dichlorophenol-indophenol (DCPIP) reduction is not detected in cell-free extracts from wild-type strain methanol- and lanthanum-grown cultures. Formaldehyde activating enzyme (Fae) is required for Ln methylotrophic growth, demonstrating that XoxF1-mediated production of formaldehyde is essential. Addition of exogenous lanthanum increases growth rate with methanol by 9–12% but does not correlate with changes to methanol consumption or formaldehyde accumulation. Transcriptomics analysis of lanthanum methanol growth shows upregulation of *xox1* and downregulation of *mxa* genes, consistent with the Ln-switch, no differential expression of formaldehyde conversion genes, downregulation of pyrroloquinoline quinone (PQQ) biosynthesis genes, and upregulation of *fdh4* formate dehydrogenase (FDH) genes. Additionally, the Ln-dependent ethanol dehydrogenase ExaF reduces methanol sensitivity in the *fae* mutant strain when lanthanides are present, providing evidence for the capacity of an auxiliary role for ExaF during Ln-dependent methylotrophy.

## Introduction

A direct link between the Ln elements and microbial metabolism has been firmly established with the discovery of PQQ-dependent alcohol dehydrogenases (ADHs), from methylotrophic bacteria, that contain a Ln atom in the active site^1–5^. Thus far, Ln-PQQ ADHs can be grouped by their phylogeny and primary substrate as either XoxF-MDHs or ExaF-type ethanol dehydrogenases (EtDHs). MxaFI MDH has been considered the canonical primary catalyst for methanol oxidation in Gram-negative methylotrophs^6,7^. The heterotetramer MxaFI contains PQQ that coordinates the calcium (Ca) ion^8–10^. The discovery that Ln is incorporated into the active site of XoxF MDH in place of Ca, allowing catalytic function, has prompted the reexamination of methanol oxidation in methylotrophic bacteria^3,5,11–18^.

To date, only a few XoxF MDHs have been kinetically characterized^1,3,4,19,20^. Phylogenetic analyses show there are at least five distinct families of XoxF MDHs^11,21^, and while it has been suggested that all XoxF MDHs may exhibit similar kinetic properties, reported data for these enzymes are currently inadequate to support such a broad characterization. In fact, recent studies have begun to identify differences in kinetic properties, cofactor usage, and pH optima of phylogenetically distinct XoxF enzymes^20,22,23^. Lack of genes encoding the dephosphotetrahydromethanopterin (H_4_MPT) pathway and catalytic properties observed for XoxF MDH from *Methylacidiphilum fumariolicum* SolV revealed the capability to produce formate as the end product of periplasmic methanol oxidation^3^. Keltjens *et al*., however, cautioned that release of formaldehyde rather than formate by XoxF MDH could occur in a “type-specific way”^11^. In support of this, transcriptomic analyses of *Methylosinus trichosporium* OB3b showed no differential regulation with Ln of genes encoding the H_4_MPT and tetrahydrofolate (H_4_F) pathways for cytoplasmic conversion of formaldehyde and formate^24^.

The genome of *M*. *extorquens* AM1 contains two *xoxF* genes, named *xoxF1* and *xoxF2*, respectively^25^. Either gene product is capable of supporting growth with methanol and exogenous Ln. XoxF1, however, is the primary methanol oxidation system for Ln-dependent methylotrophy^16^. Increased catalytic function was observed for XoxF1 purified with lanthanum (La^3+^) as a cofactor, leading to the description of the enzyme as La^3+^-dependent^2^. The only detailed kinetic analysis available for XoxF1, however, was conducted with enzyme from culture grown in the absence of Ln and only low catalytic activity was observed^26^. *In vivo* evidence is suggestive of XoxF1-catalyzed formaldehyde oxidation in starving cells fed methanol^26^. Since these studies were done in the absence of Ln, and due to the relevance of this enzyme for Ln-dependent methylotrophy and the scarcity of fundamental information available for Ln-dependent enzymes, particularly with other Ln cofactors, a detailed kinetic study of XoxF1 MDH with Ln is needed.

In addition, we reported the first Ln-dependent EtDH ExaF, showing that Ln can impact multi-carbon as well as one-carbon metabolism^5^. ExaF exhibits relatively low methanol dehydrogenase activity, but has the highest catalytic efficiency with ethanol of any reported PQQ-dependent EtDH. Additionally, *in vitro* oxidation of methanol to formate by ExaF has been reported^5^. This catalytic activity has not yet been corroborated *in vivo*.

Defining the kinetic differences of Ln-dependent MDHs and assessing the impacts they have on downstream metabolism are important steps to better understanding the implications of these metals in biology. Methanol-dependent growth in *M*. *extorquens* AM1 requires the H_4_MPT pathway to link highly reactive formaldehyde produced from methanol oxidation to the pterin carbon carrier^27^. This pathway is indispensable for growth on methanol in the absence of Ln for *M*. *extorquens* AM1, and it functions for both formaldehyde oxidation and generation of NAD(P)H and formate^28,29^. If XoxF1 exhibits catalytic properties like those of XoxF from *M*. *fumariolicum* SolV or ExaF, and produces formate from methanol oxidation, one can ask: is the H_4_MPT pathway dispensable? If so, how does the cell balance methanol oxidation with production of reduced storage compounds such as NAD(P)H needed for assimilation? If, instead, XoxF produces formaldehyde from methanol oxidation, are there any metabolic implications of Ln-dependent methylotrophy or does growth resemble canonical methylotrophy?

In this study, we biochemically characterize XoxF1 MDH from the model methylotroph *M*. *extorquens* AM1. We confirm that XoxF1 MDH is dependent on Ln metals for catalytic function; show that formaldehyde can be oxidized by pure enzyme; and demonstrate that the pure enzyme exhibits properties similar to those reported for MxaFI and other Type V XoxF enzymes. We further show that wild-type cell-free extracts from methanol plus La^3+^ grown cultures do not exhibit formaldehyde oxidation when measured by DCPIP reduction, and that the H_4_MPT pathway is required during Ln methylotrophy, providing *in vivo* evidence that XoxF1 MDH does not oxidize methanol to formate in the periplasm. Additionally, we show that ExaF EtDH reduces methanol sensitivity in a *fae* deletion mutant strain by alleviating toxic accumulation of formaldehyde, thus providing evidence of an auxiliary capacity for formaldehyde oxidation during Ln-dependent methylotrophy.

## Results

### Enzyme kinetics of XoxF1 MDH - a *bona fide* Ln-dependent methanol dehydrogenase

XoxF1 from *M*. *extorquens* AM1 is a La^3+^- and PQQ-dependent MDH and the primary oxidation system utilized for Ln-dependent growth with methanol^2,16^. Detailed kinetic parameters for the enzyme, however, have only been reported for XoxF1 purified in the absence of La^3+^ ^26^. We kinetically characterized XoxF1, a Type V XoxF, as an additional representative to the few Ln ADHs reported thus far in order to understand if representative enzymes from the same phylogenetic clade exhibit similar kinetic properties. Prior to the current study, catalytic properties of a Type V XoxF MDH have not been studied with additional Ln.

C-terminally histidine-tagged XoxF1 from *M*. *extorquens* AM1 was produced in cultures grown with methanol and either 20 μM LaCl_3_ or NdCl_3_. The protein was enriched by immobilized metal affinity chromatography (IMAC) and purified to homogeneity after histidine tag cleavage using recombinant tobacco etch virus (rTEV) protease^30,31^ (Supplementary Fig. S1). Methanol oxidation was measured by monitoring the phenazine methosulfate (PMS) -mediated reduction of DCPIP^8,16^. PQQ was detected as a prosthetic group via UV-visible spectroscopy, with a molar ratio of 1.2 mol of PQQ per mol of enzyme using a molar extinction coefficient of 9,620 M^−1.^ cm^−1^ (Supplementary Fig. S1)^5,32^. Metal content determined by inductively coupled plasma mass spectrometry (ICP-MS) showed that XoxF1-La contained La^3+^ in 1:1 molar ratio of metal to protomer.

Kinetic parameters were determined for XoxF1-La (Table 1, lines 1, 4, and 9). As kinetic parameters for XoxF1 purified in the absence of Ln have already been reported, we can compare XoxF-La to XoxF-noLn^26^. With La^3+^ bound, the *V*_max_ of methanol oxidation via XoxF1 was 380-fold greater (Table 1, lines 1 and 3**)**. The *K*_M_ for methanol was 4-fold greater for the La form of the enzyme. Combined, the changes resulted in a 91-fold increase in catalytic efficiency when La^3+^ is coordinated in the enzyme (Table 1, lines 1 and 3). Increased enzyme function with bound La^3+^ was also observed for formaldehyde and ethanol oxidation (Table 1). With formaldehyde as the substrate, the *V*_max_ increased 357-fold, the *K*_M_ increased less than 2-fold, and catalytic efficiency increased 218-fold (Table 1, lines 4 and 6). With ethanol as the substrate, *V*_max_, *K*_M_, and catalytic efficiency increased 300-fold, 45-fold, and 6-fold respectively (Table 1, lines 9 and 10). These results show a definitive increase in catalytic function of XoxF1 MDH when coordinating La^3+^, and they indicate that a change in oxidation rate is the cause of this increase. Relative to the recently characterized Type V XoxF MDH from *Methylomonas* sp. LW13, kinetic parameters of XoxF1-La with methanol were near identical (percentage identical: *V*_max_ 95%; *K*_M_ 87%; catalytic efficiency 79%)^20^.

**Table 1:** Kinetic properties of XoxF1 MDH.

Cofactor and substrate	*V*_max_ (U-mg^−1^)^§€^	*K*_M_ (mM)^€^	*k*_cat_/*K*_M_ (s^−1^ mM^−1^)^¥^
1 XoxF-La [methanol]	5.72 ± 0.13	0.044 ± 0.005	272 ± 31
2 XoxF-Nd [methanol]	2.42 ± 0.03	0.029 ± 0.002	210 ± 14
3 XoxF-noLn^ǂ^ [methanol]	0.015	0.011	3
4 XoxF-La [formaldehyde]	5.00 ± 0.07	0.096 ± 0.006	109 ± 17
5 XoxF-Nd [formaldehyde]	2.33 ± 0.05	0.133 ± 0.011	36 ± 7
6 XoxF-noLn^ǂ^ [formaldehyde]	0.014	0.065	5
7 XoxF-La (wild-type) [formaldehyde]^£^	not detected	—	—
8 ExaF-La (MDH-3) [formaldehyde]^£^	0.058 ± 0.004	—	—
9 XoxF-La [ethanol]	6.21 ± 0.12	0.067 ± 0.003	194 ± 9
10 XoxF-noLn^ǂ^ [ethanol]	0.024	0.014	4

^§^Activity was measured as the PMS-mediated reduction of DCPIP with one unit of specific activity defined as one micromole DCPIP reduced per minute (monitored at 600 nm). Inactive enzyme and no enzyme controls exhibited no activity for all enzyme preparations.

^€^Errors represent the standard deviation of the average of three independent experiments.

^¥^Errors reflect the standard deviation determined by error propagation, Z = (A ± dA)/(B ± dB).

^£^Specific activity determined from cell-free extract. Strain shown in parentheses with the primary MDH for that strain/condition shown.

^ǂ^Parameter values from Schmidt *et al*.^26^. Error values were not reported. Enzyme was purified from cells grown without exogenous Ln.

Extending the metal dependence analysis, we purified XoxF1 from cultures grown with exogenous neodymium (Nd^3+^) and kinetically characterized the enzyme with methanol and formaldehyde as substrates (Table 1, lines 2 and 5). ICP-MS metal determination of XoxF1-Nd protein resulted in a 0.5:1 Ln to protomer molar ratio. Lower metal content did not result in less bound PQQ (data now shown). Reconstitution to a 1:1 Nd to protomer ratio was unsuccessful *in vitro*. The Nd^3+^ content correlated with a ~50% reduction in *V*_max_ compared to XoxF1-La with either methanol or formaldehyde as the substrate. Relative changes in *K*_M_ values were divergent and substrate-dependent. The *K*_M_ for methanol decreased 33%, while the *K*_M_ for formaldehyde increased 39%, indicating that Ln may differentially impact MDH affinity for a substrate.

### Formaldehyde oxidation by cell-free extracts

The capacity of XoxF1-La to oxidize formaldehyde using a pure component system is in line with the assumption that XoxF MDHs oxidize methanol to formate, with formaldehyde as an intermediate and secondary substrate. However, *in vitro* activity of pure enzyme is not conclusive evidence of *in vivo* activity. To further investigate the capacity of XoxF-La to oxidize formaldehyde *in vivo* we conducted MDH assays following the PMS-mediated reduction of DCPIP, using formaldehyde as substrate with cell-free extracts of cultures grown with methanol. No activity was detected for the wild-type strain from extracts prepared from cultures grown with or without La^3+^ (data not shown). The lack of formaldehyde oxidation activity in cell-free extracts from cultures grown with La^3+^ suggests that formaldehyde is the product of methanol oxidation by XoxF1 *in vivo*. Previously, we reported that cell-free extracts of the MDH-3 mutant strain grown with methanol and La^3+^ exhibited formaldehyde oxidation activity using this assay^5^. The MDH-3 mutant strain has null mutations in *mxaF*, *xoxF1*, and *xoxF2*, and is dependent on the Ln-dependent ethanol dehydrogenase ExaF for methanol oxidation. In this study, using the same assay we measured a specific activity of 58 ± 4 nmol^.^mg^−1.^min^−1^ with extracts from the MDH-3 mutant strain grown with methanol +La^3+^ and formaldehyde as the assay substrate, confirming that ExaF can oxidize formaldehyde *in vivo*.

### Formaldehyde-activating enzyme is required for Ln-dependent growth with methanol

**Figure 1:**
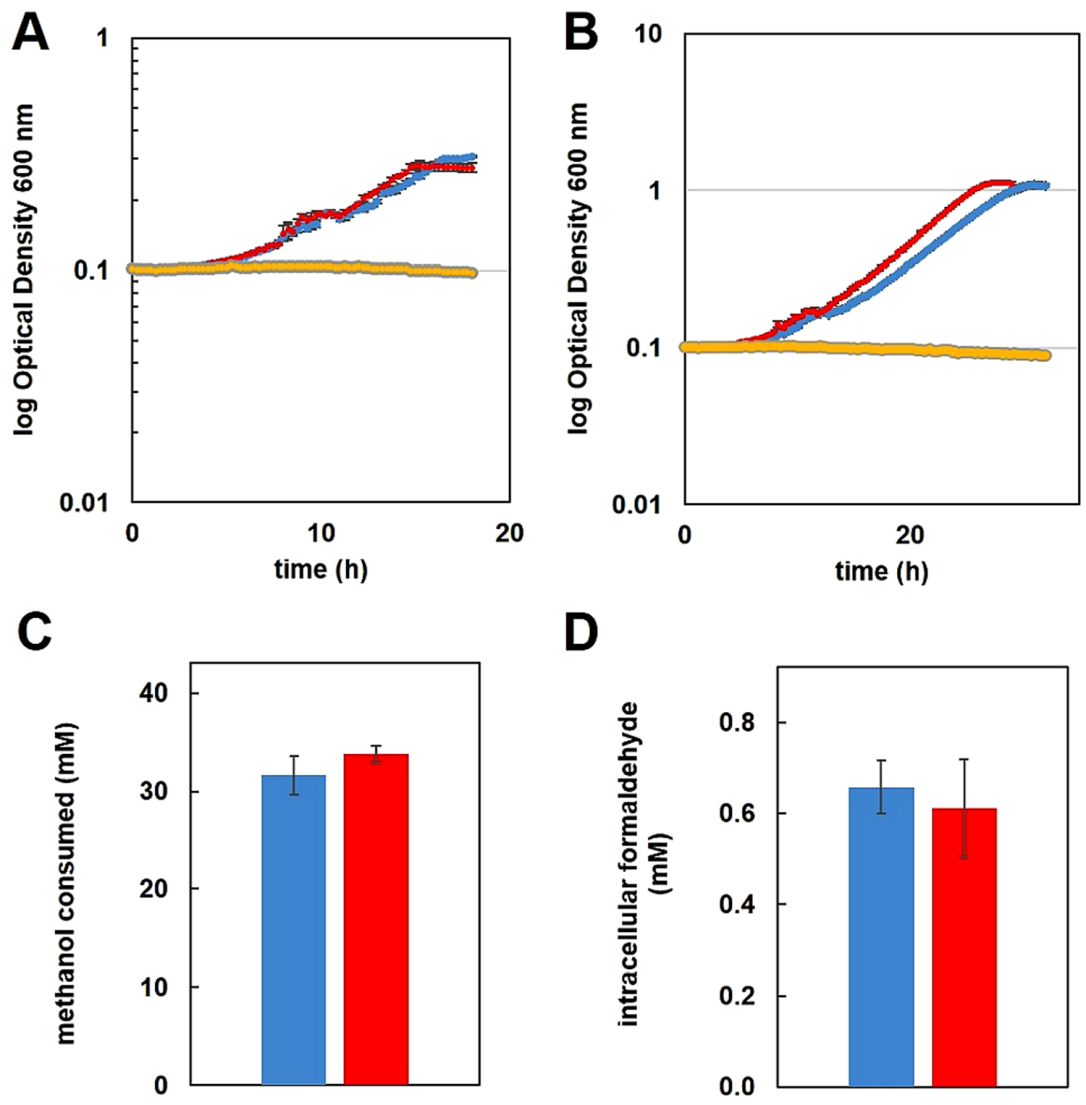
Growth of wild-type and *fae* mutant strains of *M*. *extorquens* AM1 in MP minimal medium with 15 mM **(A)** or 125 mM **(B)** methanol, in the presence [red, wild-type; gray, *fae*] or absence [blue, wild-type; yellow, *fae*] of 2 μM exogenous LaCl_3_. Cultures were grown in 48-well cell culture plates shaking at 548 rpm at 30 ^ο^C in an EpochII plate reader. Error bars represent SEM of 18 biological replicates. Not all error bars are visible due to the size of the marker representing the error. **(C)** Methanol consumption determined by HPLC analysis. Residual methanol in the growth medium at the time of harvest was subtracted from the initial substrate concentration. Values were normalized by subtracting methanol evaporated from uninoculated controls during the same growth period. Values are the mean of 4 biological replicates. **(D)** Intracellular formaldehyde concentrations determined by the Purpald assay. For substrate and metabolite measurements, cultures were grown in 50 mL minimal medium with an initial concentration of 125 mM methanol as the growth substrate and harvested in mid-exponential growth phase at OD_600_ of 1.0. The wild-type strain was grown in the absence [blue] or presence [red] of 2 μM exogenous LaCl_3_. Values represent the mean of three biological replicates. In panels C and D error bars represent the standard deviation for all biological replicates. Multiple-way analysis of variance (ANOVA) was used to determine significance of changes (p > 0.05).

Under Ln-free conditions, a formaldehyde-activating enzyme (*fae*) null mutant strain cannot grow with methanol as a carbon source due to the lack of enzymatic coupling of free formaldehyde to the carbon carrier H_4_MPT, resulting in reduced carbon flux to formate and toxic accumulation of formaldehyde^33,34^. If formaldehyde is produced by XoxF1-mediated methanol oxidation, the H_4_MPT pathway for formaldehyde oxidation should be required for La-dependent methanol growth. If XoxF1 solely produces formate as the final oxidation product of methanol, Fae activity and the H_4_MPT pathway could be dispensable during growth with Ln. We tested the *fae* mutant strain for its ability to utilize methanol as the sole substrate with and without La. No growth was observed for either condition with 15 mM methanol as the growth substrate (Fig. 1A), indicating that Fae, and by association the H_4_MPT pathway, is still needed for Ln-dependent growth on methanol. We observed the same inability of the *fae* mutant strain to grow when the methanol concentration was increased to 125 mM (Fig. 1B). Carbon flow through the H_4_MPT pathway, therefore, is required for Ln-dependent methylotrophy providing *in vivo* evidence with a growing culture that XoxF1 catalyzes the production of formaldehyde, and not formate, from methanol.

### Impact of La^3+^ on methanol growth

The effect of Ln on growth rate and yield of methylotrophic bacteria varies by report, ranging from essential for growth^3^, to having a small impact^5,18^, to having no impact^2,14,16^ on growth with methanol. Because XoxF1- and MxaFI-catalyzed methanol oxidation produces formaldehyde, one could expect that Ln-dependent methylotrophy would not differ from methylotrophy without Ln. Using 15 mM methanol as the growth substrate, the wild-type strain grew with specific growth rates of 0.207 ± 0.005 h^−1^ (+La) and 0.185 ± 0.002 h^−1^ (−La) (Fig. 1A). This change constituted a 12% increase in growth rate with exogenous La^3+^. When the concentration of methanol was increased to 125 mM, the growth rate was 9% faster for the +La condition (μ = 0.185 ± 0.004 h^−1^) relative to the −La condition (μ = 0.170 ± 0.003 h^−1^) (Fig. 1B). The increase in growth rate seen with the addition of La^3+^ was statistically significant for both methanol concentrations (*p*-value < 0.00001 by One-way ANOVA). Increasing from 15 mM to 125 mM methanol resulted in 10% and 8% reductions in growth rate for the +La and −La conditions respectively. Next, to further characterize the impact of XoxF1 MDH on methanol growth we measured both methanol consumption and intracellular formaldehyde. Neither methanol consumption nor formaldehyde accumulation changed significantly (Fig. 1C,D), indicating that a switch of MDH is not responsible for the observed change in growth rate.

### ExaF is a Ln-dependent auxiliary formaldehyde oxidation system

**Figure 2:**
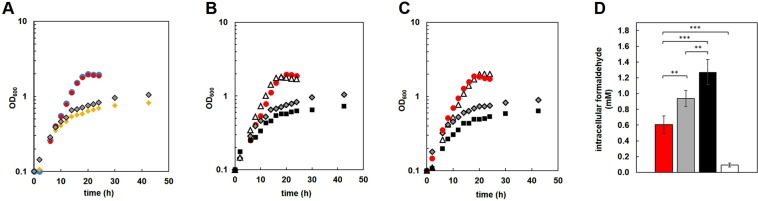
Growth of wild-type and mutant strains on succinate with methanol addition. All strains were grown in plastic 15 mL culture tubes. **(A)** Growth of wild-type and *fae* mutant strains on succinate with addition of 10 mM methanol (final concentration) after 2 h of growth. Cultures were grown in the presence (red, wild-type; gray, *fae*) or absence (blue, wild-type; yellow, *fae*) of 2 μM exogenous LaCl_3_. **(B**,**C)** Wild-type [red circles], Δ*fae* [gray diamonds], Δ*fae* MDH-3 (white triangles), and Δ*fae* Δ*exaF* (black squares) mutant strains were grown in the presence of 2 μM LaCl_3_ and methanol (panel B, 10 mM or C, 125 mM) was added after two h of growth with succinate. Time points represent three biological replicates with variances <5%. **(D)** Internal formaldehyde concentrations, determined by Purpald, for wild-type (red), *fae* (gray), *fae exaF* (black), and *fae* MDH-3 (white) mutant strains. Samples were collected at mid-exponential growth for each strain, corresponding to OD_600_ of: wild-type, 1.0; *fae* 0.4; *fae exaF*, 0.4; *fae* MDH-3, 1.0. Values represent the average of 3 biological replicates with standard deviation (error bars). One-way ANOVA was used to determine significance of changes (**p < 0.05, ***p < 0.01).

An *fae* mutant strain is able to grow with methanol in Ln-free minimal medium if succinate is included as well^29^. Under these conditions the *fae* mutant grows at a slower rate and growth arrests at a lower final OD_600_ than the wild-type strain due to accumulation of the toxic compound formaldehyde^29^. Therefore, growth phenotypic analysis under these conditions is effectively an *in vivo* methanol sensitivity assay. We investigated further the *in vivo* capacity for formaldehyde oxidation using the methanol sensitivity assay. Should either XoxF, ExaF, or both, oxidize formaldehyde, *in vivo* methanol tolerance of the strain would be expected to increase. We first measured growth of the *fae* mutant with La^3+^ and without La^3+^ using succinate and 10 mM methanol as growth substrates (Fig. 2A). Relative to the wild-type strain, the *fae* mutant strain grew more slowly and exhibited a strong reduction in growth rate after surpassing OD_600_ 0.5 in both the +La and −La conditions. After the observed downshift in growth rate, the *fae* mutant strain grew at the same rate regardless of the presence or absence of exogenous La^3+^. However, the final OD_600_ was 22% greater in the +La cultures (OD_600_ of 0.8 without La^3+^ vs 1.0 with La^3+^). This finding indicates that addition of La^3+^ to the growth medium reduces methanol sensitivity for the *fae* mutant strain, and is suggestive that *M*. *extorquens* AM1 is more tolerant of formaldehyde during Ln methylotrophy.

During growth with methanol and Ln, *M*. *extorquens* AM1 produces XoxF1and ExaF. Although both enzymes in pure form are capable of oxidizing formaldehyde, we have shown that only cell-free extracts of the MDH-3 strain (reliant on ExaF for methanol oxidation) and not the wild-type strain (dependent on XoxF1) exhibits formaldehyde oxidation activity as measured by the PMS-mediated reduction of DCPIP. Deletion of *exaF* alone does not impact growth with methanol, with or without Ln^5^, demonstrating that ExaF is not a primary methanol oxidation system. However, given the biochemical capacity of the enzyme, we investigated the possibility that ExaF may function as an auxiliary formaldehyde oxidation system in a condition where formaldehyde accumulates. We conducted the same methanol sensitivity studies using *fae exaF* and *fae* MDH-3 mutant strains with La^3+^ to specifically differentiate the activity of XoxF1 or ExaF respectively. Strains were grown in succinate medium and La^3+^ for 2 h and then 10 mM or 125 mM methanol was added. After addition of 10 mM methanol, the *fae exaF* mutant strain grew 48% slower and the maximum OD_600_ was 29% lower than for the *fae* mutant strain (Fig. 2B). Similar effects on growth were seen when the concentration of methanol added was increased to 125 mM, with a 37% reduction in growth rate and 25% reduction in final OD_600_ (Fig. 2C). These results suggested that ExaF was contributing to formaldehyde oxidation *in vivo* when the intermediate accumulates to toxic levels. The *fae* MDH-3 mutant strain, on the other hand, displayed a growth pattern resembling the wild-type strain when either 10 mM or 125 mM methanol was added to succinate growth medium (Fig. 2B,C), showing that with ExaF available the strain is not inhibited by methanol addition.

The observed lack of methanol inhibition for the *fae* MDH-3 mutant strain could be due to either increased ExaF activity, resulting in efficient conversion of methanol to formate, or low MDH activity via ExaF, limiting formaldehyde production to a sub-inhibitory concentration^5,16^. The latter explanation assumes that ExaF is producing formaldehyde from methanol oxidation, contrary to the evidence we have already shown in this study. Nonetheless, it is a possible explanation for the lack of methanol sensitivity seen with the *fae* MDH-3 mutant strain (Fig. 2B,C). Therefore, we measured both methanol consumption and internal formaldehyde concentrations during the methanol sensitivity assays. Methanol consumption was measured 10 hours after addition of 125 mM methanol, with all strains consuming 20 ± 4 mM methanol (data not shown in Fig. 2; differences among strains are insignificant by One-way ANOVA, p > 0.4). Measurement of internal formaldehyde concentrations (Fig. 2D) showed that the *fae* mutant strain accumulated 73% more formaldehyde compared to the wild-type strain. The *fae exaF* double mutant strain accumulated 26% more formaldehyde than the *fae* mutant strain, indicating that ExaF contributes to oxidation of the necessary, but toxic, intermediate *in vivo*. In comparison, the formaldehyde levels of the *fae* MDH-3 mutant strain were only 9% compared to the *fae* mutant strain. This constitutes a ~4-fold reduction in formaldehyde accumulation. Since addition of as little as 1 mM methanol is known to be inhibitory to growth of the *fae* mutant strain^29^, the lack of growth inhibition observed in the *fae* MDH-3 mutant indicates an alternative formaldehyde oxidation system is the cause of reduced internal formaldehyde. Overall, these results provide further evidence that ExaF is capable of *in vivo* formaldehyde oxidation and can function as an auxiliary Ln-dependent oxidation system to prevent inhibitory or lethal accumulation of this toxic intermediate.

**Figure 3:**
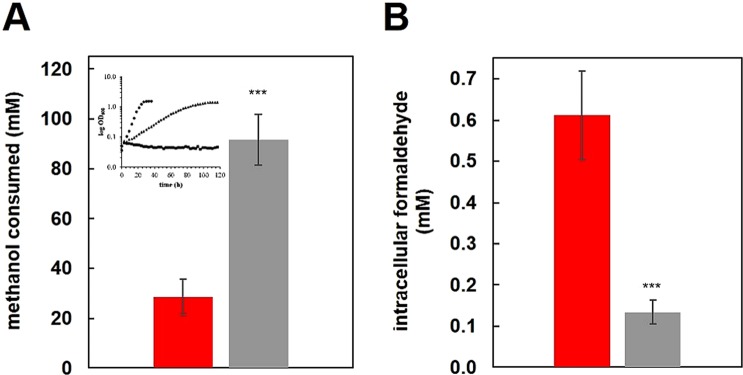
*In vivo* methanol oxidation of the MDH-3 mutant strain during Ln-dependent methanol growth. The MDH-3 mutant strain was grown on 125 mM methanol in 50 mL cultures of minimal medium with 2 μM LaCl_3_. **(A)** Methanol consumption for the MDH-3 strain (gray) determined by HPLC at a culture density of OD_600_ 1.0. **(B)** Intracellular formaldehyde (MDH-3, gray) determined by Purpald assay. For panels A and B, substrate and metabolite measurements are shown for the wild-type strain for the same growth condition (red). One-way ANOVA was used to determine significance of changes (***p < 0.01). Panel A inset shows a reference growth curve modified from Good *et al*., 2016 for the wild-type (circles, fast growth), MDH-3 (triangles, reduced growth rate), and ADH-4 (squares, no growth) with the same growth conditions^5^. The ADH-4 strain has null mutations in *mxaF xoxF1 xoxF2* and *exaF*.

We previously reported a severe reduction in growth rate for the MDH-3 mutant strain compared to the wild-type strain when both strains were grown on methanol with La^3+^ (0.042 h^−1^ and 0.152 h^−1^ respectively)^5^. It was presumed at the time that the slow growth rate of the MDH-3 mutant strain was due to the relatively high *K*_M_ for methanol as the growth substrate (5.98 mM). However, upon further review, the amount of methanol added should not have been limiting, and the results reported herein show that ExaF is capable of *in vivo* formaldehyde oxidation during methanol growth. Therefore, an alternative explanation for the methanol growth defect observed for the MDH-3 mutant strain is that ExaF produces formate, and not formaldehyde, from methanol oxidation. To further investigate this possibility, we measured methanol consumption (Fig. 3A) and internal formaldehyde levels (Fig. 3B) when MDH-3 is growing only on methanol. Under the same growth conditions, the MDH-3 mutant strain consumed 3-fold more methanol than the wild-type strain to obtain the same culture density. This correlated with a 3–3.5-fold reduction of intracellular formaldehyde in the MDH-3 mutant strain. Together, these results provide corroborating evidence of ExaF-catalyzed formaldehyde oxidation *in vivo*.

### Methanol and formate oxidation and PQQ synthesis genes are differentially regulated by Ln

Our results indicate that the switch from MxaFI MDH to XoxF1 MDH during Ln-dependent methylotrophy does not have a profound impact on methanol oxidation in the wild-type strain. Nonetheless, we observed a statistically significant increase in growth rate with methanol and La^3+^ for the wild-type strain. To get further insight into changes in methylotrophic metabolism due to the presence of Ln, RNA transcript profiles of cultures grown with and without exogenous La^3+^ were compared using RNA-seq transcriptomics. Using a cutoff of |log_2_| > 1 and a false discovery rate of <0.15, we observed 116 differentially expressed genes (complete RNA-seq analysis is available in Supplementary Table S1**)**. Differentially expressed genes of particular interest to methylotrophy are shown in Table 2. In the presence of La^3+^ the genes *xoxF*, *xoxG*, and *xoxJ* encoding the Ln-dependent MDH, the predicted cognate cytochrome *c*_*L*_, and a MxaJ-like protein, were all significantly upregulated (16–21 fold). Notably, neither *exaF* nor *xoxF2* were upregulated. Lack of differential expression is consistent with the observation that a single mutation in either gene does not result in a growth defect with methanol and La^3+^. In congruence with the Ln switch reported for methanol oxidation in methylotrophic bacteria^14,16,18^, the entire *mxa* gene cluster, encoding the Ca-dependent MDH and accessory genes, was highly downregulated, (8–256-fold). The genes encoding *mxbDM*, a two-component system necessary for the expression of the *mxa* gene cluster and repression of the *xox1* genes^35^, was also downregulated ~3–4-fold. Interestingly, the *mxcQE* two-component system, also required for MDH expression and *mxbDM* induction, was not differentially expressed. Several genes important for PQQ biosynthesis and processing were downregulated as well (3–13 fold), even though XoxF1 is a PQQ-dependent dehydrogenase. These latter two observations parallel similar expression responses reported for *M*. *aquaticum* 22 A^36^.

**Table 2:** Differentially expressed genes and annotated function of *M*. *extorquens* AM1 grown on methanol with or without 2 µM La^3+^ (FDR: false discovery rate <0.15, |log_2_| fold change +La:−La > 1.0). Complete differential expression analysis is available in Supplementary File S1.

function	locus tag	log_2_ fold change	FDR
Xox-type MDH	*xoxF1* (META1p1740)	4.4	1.26E-02
	*xoxG* (META1p1741)	4.1	1.60E-04
	*xoxJ* (META1p1742)	4.0	1.16E-06
Formate dehydrogenase	*fdh4B* (META1p2093)	2.2	9.91E-03
	*fdh4A* (META1p2094)	2.1	1.25E-02
ABC transporter	(META1p1737)	3.1	8.08E-06
	(META1p1738)	2.9	2.48E-05
	(META1p1739)	2.7	5.78E-04
Mxa-type MDH	*mxaB* (META1p4525)	−3.4	1.98E-09
	*mxaH* (META1p4526)	−3.1	4.53E-07
	*mxaE* (META1p4527)	−4.9	2.02E-14
	*mxaD* (META1p4528)	−6.2	1.12E-26
	*mxaL* (META1p4529)	−5.4	2.12E-17
	*mxaK* (META1p4530)	−5.7	1.18E-19
	*mxaC* (META1p4531)	−5.1	4.18E-12
	*mxaA* (META1p4532)	−4.3	8.67E-11
	*mxaS* (META1p4533)	−5.3	6.81E-15
	*mxaR* (META1p4534)	−7.0	1.44E-22
	*mxaI* (META1p4535)	−7.8	1.96E-08
	*mxaG* (META1p4536)	−7.4	1.58E-15
	*mxaJ* (META1p4537)	−7.5	2.00E-18
	*mxaF* (META1p4538)	−8.0	7.52E-09
Two-component regulatory system	*mxbM* (META1p1752)	−1.4	1.17E-01
	*mxbD* (META1p1753)	−2.0	1.33E-03
PQQ biosynthesis	*pqqE* (META1p1748)	−1.8	3.20E-02
	*pqqCD* (META1p1749)	−2.0	1.07E-02
	*pqqB* (META1p1750)	−1.7	2.80E-02
	*pqqA* (META1p4628)	−3.7	7.17E-05
	*pqqA* (META1p4629)	−3.4	9.67E-07

Significantly, neither genes encoding the formaldehyde oxidizing H_4_MPT pathway nor the H_4_F pathway for formate conversion were differentially expressed, consistent with our biochemical, genetic and phenotypic studies indicating that XoxF1 produces formaldehyde *in vivo*. Similar observations were noted for *M*. *trichosporium* OB3b, a serine cycle methanotroph^15^. The *M*. *extorquens* AM1 genome encodes at least four distinct FDHs: *fdh1*, *fdh2*, *fdh3*, and *fdh4*. The four FDHs are redundant under standard laboratory conditions, with *fdh4* being the only single knockout that has a measurable impact on growth^37^. Only the genes encoding Fdh4 were differentially expressed, being upregulated 4-fold. The genes encoding the assimilatory serine cycle and the EMC pathway were not differentially expressed.

Overall, the gene expression profile of *M*. *extorquens* AM1 during La-dependent methylotrophy show the regulatory switch from MxaFI MDH to XoxF MDH and significant changes to expression of PQQ synthesis. These observations parallel prior studies targeting the impact of Ln on gene expression in methylotrophs^14,15,18,36^ A noteworthy difference, however, is the novel pattern of *fdh4* gene expression.

## Discussion

The study of Ln-dependent enzymes is a growing field of study. XoxF1 MDH from *M*. *extorquens* AM1 is a representative enzyme from the Type V clade of XoxF MDH. Recently, kinetic parameters of a Type V XoxF MDH from *Methylomonas* sp. LW13 were reported and found to be very similar to reported values of MxaFI enzymes^11,20^. Kinetic characterization of XoxF1 reveals it to be as catalytically efficient as the Type V XoxF from *Methylomonas* sp. LW13^20^. Pure XoxF1 exhibits similar catalytic efficiency with either methanol or formaldehyde as the substrate, differentiating it from the LW13 Type V XoxF, for which a 10-fold reduction in efficiency was observed with formaldehyde. Representative enzymes from the same clade, therefore, cannot be assumed to share similar kinetic properties, necessitating the study of individual enzymes from organisms of interest. The remarkable catalytic efficiency with methanol observed for the Type II XoxF MDH from *M*. *fumariolicum* SolV is the result of the exceptionally low *K*_M_ for methanol as the substrate^3^. Intriguingly, the reduced catalytic efficiency of XoxF1 from strain AM1 relative to that from strain SolV is attributable to its greater *K*_M_ for methanol. It is notable that XoxF from strain SolV was purified and initially characterized with a mixture of Ln. When the same enzyme was characterized with only europium (a heavy Ln) the catalytic efficiency was greatly reduced, demonstrating that the type of Ln coordinated may impact activity. Consistent with this observation, even though ~50% of XoxF1 purified with Nd^3+^ was in the apoprotein form, the *K*_M_ of methanol for XoxF1-Nd was ~2-fold less than that of XoxF1-La. This observation suggests that if the enzyme were fully loaded with Nd^3+^ the efficiency would be greater. In a recent study by Lumpe *et al*., it was shown that light Ln increase catalytic efficiency, while heavy Ln had the opposite effect^23^. Further detailed biochemical analyses with more representatives of all XoxF clades using various Ln cofactors are needed to fully understand how these metals impact kinetic properties. Detailed structural and/or biochemical studies may be necessary to pinpoint the features or mechanisms responsible for differing oxidation end products among XoxF MDH families^22^. Nonetheless, the current study contributes to our limited understanding of the impact of Ln on the catalytic properties of XoxF MDH.

Despite the capacity of pure XoxF1 to oxidize formaldehyde *in vitro*, we show several lines of evidence that this activity does not occur *in vivo*. First, we were unable to detect formaldehyde oxidation in wild-type cell-free extracts from cultures grown on methanol with La^3+^ using an established dye-linked enzymatic assay. Second, the *fae* mutant strain did not grow on methanol, confirming that formaldehyde is produced at levels sufficient to inhibit growth for Ln-dependent methylotrophy in *M*. *extorquens* AM1. Third, we did not observe expression changes for H_4_MPT pathway genes, consistent with this pathway being utilized during Ln methylotrophy. Fourth, methanol consumption and internal formaldehyde concentration measurements together constitute an *in vivo* assay for methanol oxidation activity. We did not observe a significant change in either methanol consumption or intracellular formaldehyde concentration for Ln-dependent methanol growth. Our conclusion stands in contrast to prior work that has suggested XoxF1 oxidizes formaldehyde *in vivo*. Schmidt *et al*. 2010 observed reduced formate production in *xoxF1* mutant cells that were first grown in the absence of Ln, starved, and then fed formaldehyde^26^. However, this observation was made prior to the knowledge that XoxF1 apoprotein is required for production of MxaFI^35^. Disrupting x*oxF1*, therefore, implies there is no production of XoxF1 or MxaFI, confounding the interpretation that XoxF1 oxidizes formaldehyde *in vivo*. Further, these studies were performed in the absence of Ln, and therefore cannot be directly compared.

Additionally, *M*. *extorquens* AM1 has the genetic potential to produce two other enzymes that could conceivably produce formaldehyde from methanol oxidation during Ln-dependent growth. We have shown that expression of neither *xoxF2* nor *exaF* is significantly upregulated with La^3+^, and neither single knockout mutant exhibits a measurable growth defect with methanol and La^3+^. These results indicate that XoxF2 and ExaF are not involved in formaldehyde production in the wild-type strain^16^. ExaF has been shown to support Ln-dependent methanol growth, resulting in a severe reduction in growth rate^5^. We have also shown evidence that ExaF is capable of oxidizing formaldehyde *in vivo*. This activity thus far has only been observed in conditions where formaldehyde accumulates (*fae* mutant strain) or in a genetic background where *exaF* is likely to be upregulated (MDH-3). In retrospect, the reduction in growth rate observed for the MDH-3 mutant with methanol as the growth substrate is likely generated, at least in part, to periplasmic oxidation of methanol to formate. The oxidation of formaldehyde to formate via the H_4_MPT dependent pathway produces NAD(P)H, whereas the oxidation of methanol to formate in the periplasm would involve two subsequent two electron transfers from MDH to a terminal oxidase (via cytochromes *c*_L_, *c*_H_, and *aa3* complex) and would not produce NAD(P)H^38^. Therefore, periplasmic production of formate from methanol could have substantial impacts on both catabolic and anabolic processes^11^.

XoxF1, therefore, remains the most parsimonious source of formaldehyde from Ln-dependent methanol oxidation *in vivo* for the wild-type strain. This conclusion is substantiated further by similar measurements for methanol consumption and internal formaldehyde levels comparing Ln methylotrophy to canonical methylotrophy. While we did observe an increase in growth rate, the lack of a dramatically different growth phenotype, such as that observed with the MDH-3 mutant, for the wild-type strain correlates with the evidence for XoxF1-catalyzed methanol oxidation to formaldehyde *in vivo*. In light of this, *M*. *extorquens* AM1 could serve as a model organism for comparing two distinct modes of methylotrophy: metabolism with oxidation of methanol to formaldehyde, and metabolism with periplasmic oxidation of methanol to formate.

Our RNA-seq analysis shows clear activation of the Ln switch, consistent with other gene expression studies of methylotrophs grown in the presence of Ln^14,15,18,36^. Since methanol oxidation via XoxF1 is not the cause of the Ln-dependent increase in growth rate with methanol, it suggests that changes to downstream metabolism could play a role. We observed a novel pattern of *fdh* regulation – specifically the up-regulation of *fdh4* during Ln-methylotrophy. Formate is a major branchpoint of methylotrophy in *M*. *extorquens* AM1^34^, and as such even subtle changes to oxidation rates could impact NADH production and/or assimilation. While an intriguing possibility, further experimentation is needed to confirm that the formate branchpoint is indeed differentially regulated during Ln-methylotrophy and is beyond the scope of this study. Additionally, the expression of PQQ synthesis genes was downregulated in the presence of Ln. This regulatory trend is in agreement with the recent study comparing gene expression patterns in *M*. *aquaticum* strain 22 A^36^. Why PQQ synthesis is downregulated with the upregulation of Ln-dependent PQQ ADHs in the presence of Ln is unknown. Activities of XoxF1 and ExaF do not appear to be impacted by the downregulation of PQQ synthesis genes, suggesting that any decrease in PQQ pools is not severe enough to restrict formation of holoenzyme. PQQ is a known plant growth promotion factor^39.^ It is possible that restricting PQQ plays a regulatory role, either with other enzymes of the microbe, the plant, or both. Further studies of PQQ in Ln methylotrophy are needed to determine the regulatory role of this molecule. Finally, it is noteworthy that we did not observe upregulation of the gene encoding the Ln binding protein lanmodulin^40^. While lanmodulin has been shown to bind Ln, its role in Ln metabolism, if any, is still unknown^40,41^.

*Methylobacterium* strains are associated with the plant phyllosphere, a dynamic environment where competition for volatile organic substrates, such as methanol and ethanol, is expected to be high^42–44^. Ln ADH systems such as XoxF and ExaF could function together to maintain stability of metabolism in an environment with large fluctuations in substrate availability. Such complementarity of oxidation systems, in both primary and secondary substrates, may further explain the large number of bacteria replete with redundant enzymatic systems. It has been speculated that redundant, cofactor-dependent oxidation systems may allow for effective growth under differing metal limitation. Ln, therefore, may cause a shift in metabolic strategy to one that is more tolerant of formaldehyde, although this hypothesis needs further evidence to be confirmed^45^. The underlying metabolic tradeoffs of such a switch are still unknown. In addition, the limited availability of Ln in natural environments may generate niches where there is an incomplete switch between Ca and Ln methylotrophy such that both MxaFI and XoxF systems operate simultaneously. Studies have begun to characterize such a hybrid regulatory state, showing dual expression of *mxa* and *xox* promoters under certain growth conditions^16,46^. Further characterization of simultaneous XoxF-MxaF driven metabolism is an area open for investigation.

Finally, the phenotypes, methanol consumption and formaldehyde measurements presented, including the lack of XoxF-dependent formaldehyde oxidation activity in Ln-grown cell-free extracts of the wild-type strain, indicate the existence of a mechanism for inhibiting XoxF1 oxidation capacity to generate formate. Discovery and characterization of the mechanism will have major implications in the regulation of XoxF MDH.

## Materials and Methods

### Bacterial strains and cultivation

Strains and plasmids used in this study are listed in Table 3. *Escherichia coli* strains were maintained on solidified (1.5% wt/vol agar) Lysogeny broth^47^ (BD, Franklin Lakes, NJ) at 37 °C with kanamycin added to a final concentration of 50 μg/mL. *M*. *extorquens* AM1 strains were grown in minimal salts medium^48^ prepared in new glassware that had not been exposed to Ln. 3 mL cultures were grown in round-bottom polypropylene culture tubes (Fisher Scientific, Waltham, MA, USA) at 30 °C, shaking at 200 rpm on an Innova 2300 platform shaker (Eppendorf, Hamburg, Germany), with succinate (15 mM) or methanol (125 mM) as the growth substrate, and subcultured into fresh media (volume and substrate defined by the experiment; see below). LaCl_3_ was added to a final concentration of 2 μM or 20 μM when indicated. When necessary, kanamycin was added to a final concentration of 50 μg/mL. For methanol tolerance studies, methanol was added to a concentration of 10 mM or 125 mM.

**Table 3:** Strains and plasmids used in this work.

Strain or plasmid	Description	Reference
**Strains**		
***Escherichia coli***		
DH5α	electrocompetent cloning strain	Invitrogen
S17-1	conjugating donor strain	^56^
***Methylobacterium extorquens***		
AM1	wild type; rifamycin-resistant derivative	^57^
*exaF*	Δe*xaF*	this work
*fae*	Δ*fae*	^29^
*fae exaF*	Δ*fae* Δ*exaF*	this work
*fae* MDH-3	Δ*fae* Δ*mxaF* Δ*xoxF1* Δ*xoxF2*	this work
MDH-3	Δ*mxaF* Δ*xoxF1* Δ*xoxF2*	^16^
ADH-4	Δ*mxaF* Δ*xoxF1* Δ*xoxF2* Δe*xaF*	this work
**Plasmids**		
pCM157	*cre* expression plasmid, Tc^R^	^58^
pRK2013	helper plasmid, IncP *tra* functions, Km^R^	^59^
pLB01	Km^r^, *P*_*xox1*_*-xoxF1-*hexahistidine tag, Xa site	^5^
pNG284	pLB01 with TEV cleavage site	this work
pHV24	pCM184:*exaF*; donor for *exaF*::Km	^5^
pHV2	SacB-based allelic exchange *fae* donor; Km^R^	E. Skovran

### Plasmid and strain construction

Plasmid pLB01 was constructed for producing hexahistidine-tagged XoxF1 with a Factor Xa protease cleavage site^5^. For this work, the Factor Xa cleavage site in pLB01 was substituted with the rTEV protease cleavage site^30^ to generate pNG284 using the following primers: pLB01 vector backbone; forward GCCGAACAACGGATTGGAAGTACAGGTTCTCCATCATCACCATCACCATAATTGTC, reverse CAGCTCACTCAAAGGCGGTAATAC; *xoxF1* allele insert; forward, CGTATTACCGCCTTTGAGTGAGCTGCTGAATTTAGCAGGCAAGTTTCCTG, reverse, GATGGAGAACCTGTACTTCCAATCCGTTGTTCGGCAGCGAGAAGAC. Primers were designed to generate PCR products with 20 bp homologous overlapping regions between backbone and insert fragments. The backbone forward primer and insert reverse primer were designed to contain the rTEV protease cleavage site. Vector backbone and allele insert PCR products were assembled using *in vivo* gap repair assembly^49,50^. Briefly, *E*. *coli* DH5α electrocompetent cells were transformed via electroporation with ~150 ng of backbone and insert PCR products. Transformed cells were incubated at 37 °C for ~1 h outgrowth and then plated on LB plates with kanamycin to select for transformants. Deletion mutant strains were constructed as reported^5^.

### Phenotypic analyses

For comparison of growth among strains, all media were prepared in new glassware and all cultures in new polypropylene culture tubes to minimize the likelihood of Ln contamination. Initial cultures were grown in succinate minimal media and centrifuged at 10,000 × *g* at room temperature for 2 min. Spent culture medium was removed, and cells were washed twice with fresh carbon-free culture medium before resuspension in fresh carbon-free culture medium. Growth phenotypes of wild-type *M*. *extorquens* AM1 were compared using a BioTek EpochII microplate reader (BioTek, Winooski, VT) following the procedures described in Delaney *et al*. 2013 with slight modifications^48^. Briefly, 650 μL of growth medium with methanol, with or without 2 μM LaCl_3_, were inoculated to a starting optical density (OD) of 0.1. Cultures were shaken at 548 rpm at 30 °C and the OD at 600 nm was monitored over time. OD_600_ measurements were fitted to an exponential model for microbial growth using CurveFitter (http://www.evolvedmicrobe.com/CurveFitter/). Growth curves were reproducible for 18 replicates from 3 independent experiments for each substrate concentration (Supplementary Fig. S3). Growth rates were calculated using >15 data points for 15 mM methanol growth curves and >35 data points for 125 mM methanol growth curves to determine the line of best fit using an exponential model with a semi-log plot of OD_600_ vs. time. R^2^ values for all lines of best fit were >0.992.

*M*. *extorquens* strains were tested as previously described for their sensitivity to methanol^29^. Starter cultures were grown overnight with succinate in polypropylene tubes. Three-mL cultures of minimal medium with succinate in polypropylene tubes were inoculated from starter cultures to a starting OD_600_ of 0.1 and shaken continuously at 200 rpm and 30 °C. After 2 h, solutions of methanol and/or water was added (total of 100 µL) to achieve final concentrations of 0 mM, 10 mM, or 125 mM methanol. After addition of methanol, cultures were continuously shaken and growth was monitored by measuring OD_600_ every 2 h for 24 h using an Ultraspec 10 cell density meter (Amersham Biosciences, Little Chalfont, UK). Methanol inhibition studies were performed in triplicate for each condition.

### RNA-seq transcriptomics

50 mL cultures were grown in shake flasks with or without LaCl_3_ to an OD_600_ of 0.7 that correlated with mid-exponential growth. Total RNA samples were procured, and quality was verified as previously described^51^. rRNA depletion, library preparation, and Illumina Hi-Seq sequencing were performed by the Michigan State University Research Technology Support Facility Genomics Core. Libraries were prepared using the TruSeq Stranded Total RNA kit (Illumina, San Diego, CA), with Ribo-Zero Bacteria used for rRNA depletion (Epicentre, Madison, WI). All replicates were sequenced on an Illumina HiSeq. 2500 using a multiplex strategy with 50 bp single-end reads with a target depth of >30 million mapped reads. Base calling was done by Illumina Real Time Analysis (RTA) v1.18.64 and the output of RTA was demultiplexed and converted to a FastQ format with Illumina Bcl2fastq v1.8.4. The filtered data were processed using SPARTA: Simple Program for Automated for reference-based bacterial RNA-seq Transcriptome Analysis^52^. Final abundances were measured in trimmed mean of M values (TMM).

### Methanol consumption measurements

Shake flasks were cleaned of potential Ln contamination by repeatedly growing an *mxaF* mutant until the strain no longer grew^16^. Then, flasks containing 50 mL of minimal medium with 125 mM methanol were inoculated with succinate-grown starter cultures. Cultures were grown shaking continuously at 200 rpm and 30 °C to OD_600_ of 1.0, corresponding to mid-exponential growth. The cultures were then transferred to 50 mL conical tubes (Fisher Scientific, Waltham, MA, USA) and cells were pelleted using a Sorvall Legend X1R centrifuge (Thermo Fisher Scientific, Waltham, MA) at 4,700 × *g* at 4 °C for 10 min. One mL of supernatant was centrifuged at room temperature for 25 min at 15,500 × *g* and transferred to a clean glass HPLC vial for immediate analysis. Samples were analyzed using a Shimadzu Prominence 20 A series high-pressure liquid chromatography system with an SPD-20A UV-VIS detector (Shimadzu, Kyoto, Japan) and a BioRad Aminex HPX-87H organic acids column 300 × 7.8 mm with a 9 µm particle size (BioRad, Hercules, California, USA). An isocratic flow of 5 mM HPLC grade sulfuric acid in Nanopure water was used as the mobile phase at 0.6 mL/min. Peak areas were compared with a standard curve to determine methanol remaining in the media. Due to the volatility of methanol, consumption values were normalized by subtracting methanol concentrations of samples taken from uninoculated control flasks at the same time.

### Intracellular formaldehyde and formate measurements

To determine internal concentrations of formaldehyde, cells were resuspended in 2 mL 25 mM Tris-HCl pH 8.0 with 150 mM NaCl and disrupted using a One Shot Cell Disruptor set to 25 PSI (Constant Systems, Ltd., Daventry, UK). Cell lysates were centrifuged at 15,500 *g* for 5 min at 4 °C. The supernatant was transferred to a clean 1.5 mL tube and the formaldehyde concentration was determined using the Purpald assay^53^, measuring absorbance at 550 nm using a BioTek EpochII microplate reader (BioTek, Winooski, Vermont, USA). For internal concentration estimation, a dry weight of 0.278 g/L at 1 OD_600_ unit was used, as previously reported^34^, with a cell volume of 36 µL/mg dry weight, based on an average cell size of 1 by 3.2 μm^54^ and an average of 4 × 10^8^ cells/mL at 1 OD_600_ unit^55^.

### Protein purification and analysis

2.8-L shake flasks were cleaned for Ln contamination as described above. Cultures of wild-type *M*. *extorquens* AM1 harboring pNG284 were grown with methanol and 20 μM LaCl_3_ to OD_600_ ~6. XoxF1 was purified by IMAC and processed, including determination of metal content by ICP-MS and PQQ content by UV-visible spectroscopy, as reported^5^. The absorbance spectrum of the enzyme was determined in a 1-cm-path-length cuvette at room temperature using a UV-2600 UV-Vis spectrophotometer (Shimadzu, Columbia, MD). PQQ concentration and content were calculated using the molar absorption coefficient of 9,620 M^−1^·cm^−1^ ^32^. Ln content was determined by ICP-MS at the Michigan State University Laser Ablation ICP-MS Facility using a Thermo Fisher Scientific ICAP Q ICP-MS (Waltham, MA) in collision cell mode.

### Methanol dehydrogenase activity assay and enzyme kinetics

Methanol dehydrogenase activity was measured by monitoring the PMS-mediated reduction of DCPIP according to Anthony and Zatman, with modifications reported in Vu *et al*.^8,16^. Using these modifications, there was little to no endogenous reduction of DCPIP without addition of methanol. Inactive enzyme controls were prepared by heat denaturation at 95 °C for 10 minutes. The assay parameters, therefore, were suitable for determining kinetic constants for XoxF1. Kinetic constants were determined by measuring enzyme activity with a range of substrate concentrations. Data were fitted in GraphPad Prism 6 (GraphPad Software, San Diego, CA) according to the Michaelis-Menten equation using non-linear regression. Values reported are the averages of 3 independent experiments, each with at least 3 technical replicates. Formaldehyde oxidation activity was measured using the same assay condition, but replacing methanol with 0.5 or 5 mM formaldehyde (final concentration). Formaldehyde was prepared, immediately before conducting the assays, by hydrolysis by heating paraformaldehyde in a sealed vial.

## Supplementary information

Supplementary File

Supplementary File 2

## Data Availability

The datasets generated and analyzed during the current study are in the Gene Expression Omnibus repository (https://www.ncbi.nlm.nih.gov/geo/ion), accession number: GSE125593.
