# Supplementary material for: Contrasting in vitro and in vivo methanol oxidation activities of lanthanide-dependent alcohol dehydrogenases XoxF1 and ExaF from Methylobacterium extorquens AM1
Source: Sci Rep. 2019 Mar 12;9:4248. doi: 10.1038/s41598-019-41043-1 (PMC6414531; doi:10.1038/s41598-019-41043-1)

supplementary information submitted to Scientific Reports

Contrasting *in vitro* and *in vivo* methanol oxidation activities of lanthanide-dependent alcohol dehydrogenases XoxF1 and ExaF from *Methylobacterium extorquens* AM1

Nathan M. Good[1], Riley S. Moore[1], Carly J. Suriano[1], N. Cecilia Martínez-Gómez[1*]

[1] Martínez-Gómez laboratory, Department of Microbiology and Molecular Genetics, Michigan State University, MI, USA

*** Correspondence:**
N. Cecilia Martínez-Gómez

mart1754@msu.edu

Figure S1 Purification and UV-visible spectroscopy of Ln-dependent XoxF1 MDH. **(A)** SDS-PAGE gel analysis of XoxF1 MDH purified with lanthanum (La^{3+}) cofactor. Gel lanes are numbered as follows: 1, protein molecular marker (in kDa); 2, cell-free extract; 3, column wash; 4-8 elution fractions; 9 untagged XoxF1-La. **(B)** SDS-PAGE gel analysis of XoxF1 MDH purified with neodymium (Nd^{3+}) cofactor. Gel lanes are numbered as follows: 1, protein molecular marker (in kDa); 2, untagged XoxF1-Nd; 3-5 elution fractions; 6, column wash; 7 cell-free extract. **(C)** PQQ was detected as the prosthetic group in XoxF1-La by its UV-visible spectrum, with a peak absorbance at 355 nm for enzyme at a concentration of 3.5 mg/ml in 25 mM Tris-HCl, pH 8.0.

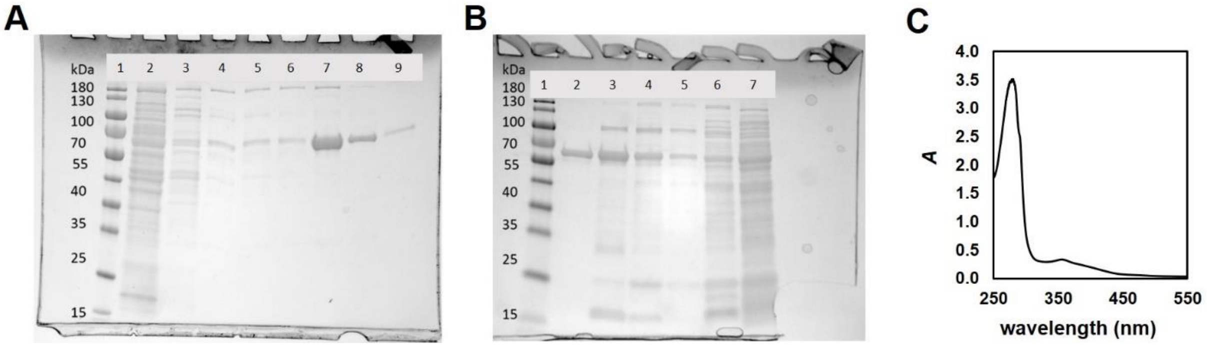

Figure S2 Biochemical characterization of Ln-dependent XoxF1 MDH. XoxF1 was purified and characterized with La (circles) or Nd (squares) as the metal cofactor. Michaelis-Menten plots using non-linear regression were generated using GraphPad Prism software (GraphPad Software, San Diego, CA). *V* vs. *S* is shown for purified XoxF1 with methanol (A), formaldehyde (B), and ethanol (C) as substrates. Plot data points represent the mean of triplicate independent measurements with errors representing the standard deviation.

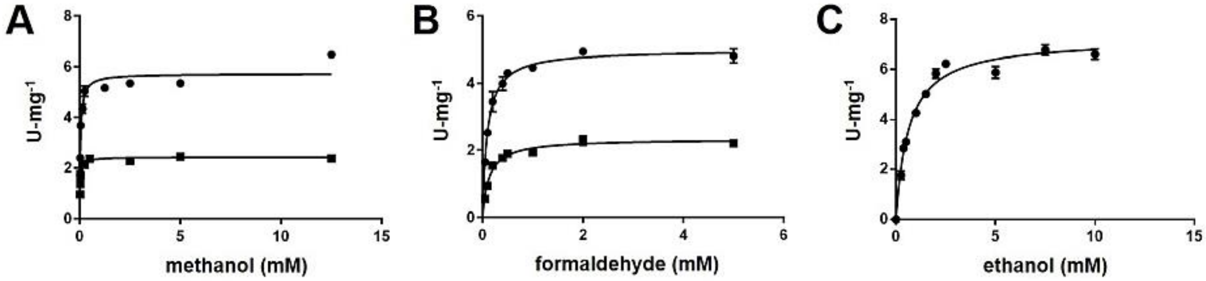

Figure S3 Cluster analysis of growth rates of wild-type *M. extorquens* AM1 grown in minimal medium with methanol. Red data points are for cultures grown with 2 µM La. Blue data points are for cultures grown without the inclusion of La. Panel A is growth rates with 15 mM methanol as the growth substrate. Panel B is growth rates with 125 mM methanol as the growth substrate. Data points reflect 18 replicates from 3 independent experiments. Growth rates for each condition are similar within 2%, and differences between +La and –La are statistically significant (p-value < 0.00001 by One-way ANOVA for both substrate concentrations).

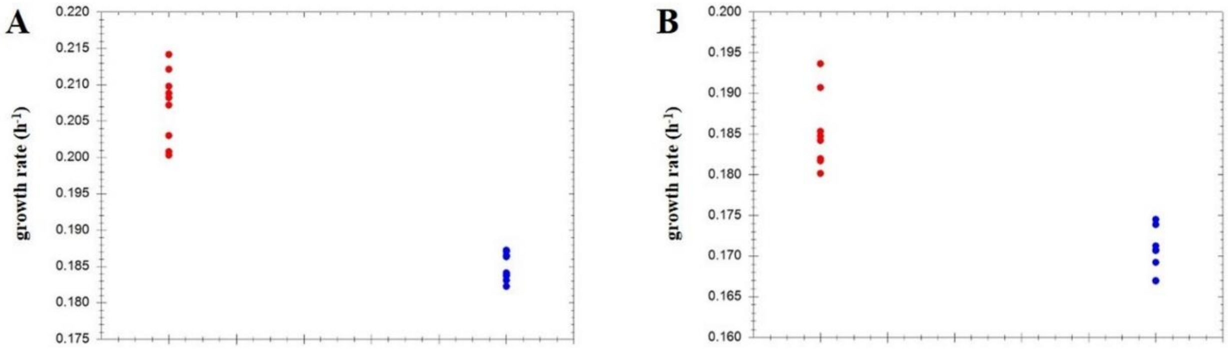

Figure S4 Multidimensional scaling plot for log_2-transformed expression data for all coding sequences. La refers to datasets for cultures grown with 125 mM methanol and 2 µM La. noLa refers to datasets for cultures grown with 125 mM methanol without addition of La.

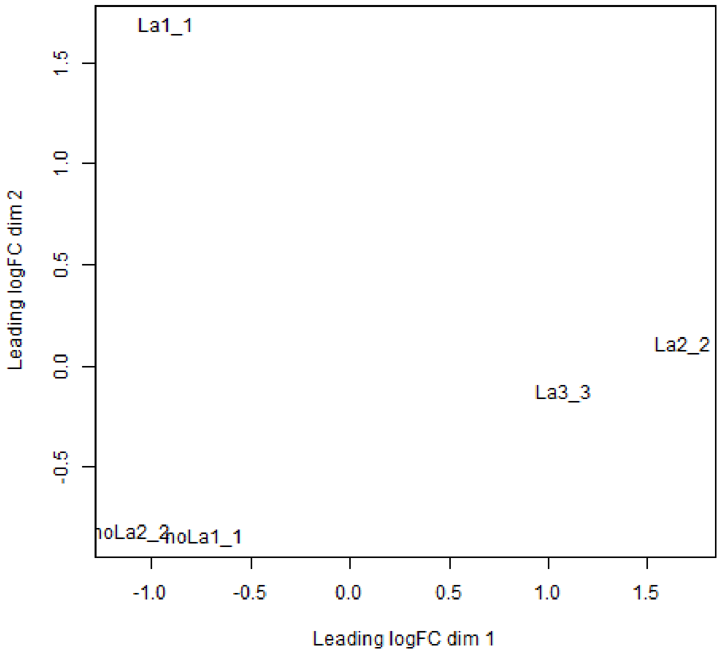

Supplement: Supplementary file 1 — Supplementary File [file 41598_2019_41043_MOESM1_ESM.pdf]
